# A Case of Pituitary Apoplexy and Cavernous Sinus Syndrome during Hemodialysis

**DOI:** 10.1155/2023/3183088

**Published:** 2023-04-25

**Authors:** Yusra Jamal, Yudi Camacho, Simon Hanft, Patrick Chiarolanzio, Michael D. Goldberg, Jamie A. Mullally

**Affiliations:** ^1^Division of Endocrinology, Department of Medicine, Westchester Medical Center, New York, USA; ^2^Department of Neurosurgical Oncology, Westchester Medical Center, New York, USA; ^3^Department of Radiology, Westchester Medical Center, New York, USA

## Abstract

**Background:**

Pituitary apoplexy (PA) is a clinical syndrome of pituitary hemorrhage or infarction and can result in hypopituitarism as well as compression of adjacent brain structures. Visual loss occurs frequently, as a result of tumor expansion and compression of the optic chiasm and optic nerves. Additionally, with pituitary tumor invasion into the fixed space of the cavernous sinus, compression of multiple cranial nerves can result in cavernous sinus syndrome (CSS). We describe a case of an undiagnosed pituitary tumor manifesting as abrupt PA with CSS during hemodialysis (HD). *Clinical Case*. A 77-year-old male with end-stage renal disease (ESRD) presented with acute onset of severe headache, decreased vision, ophthalmoplegia of the left eye, and hypotension during HD. MRI of the brain revealed a 2.5 cm pituitary adenoma with acute hemorrhage, compression of the left prechiasmatic optic nerve, and invasion into the left cavernous sinus (CS). The hormonal profile was consistent with multiple pituitary hormone deficiencies. The patient was treated with glucocorticoids and underwent transsphenoidal resection of the tumor. He had an uneventful postoperative hospital course, and his left visual acuity stabilized, although there was no immediate improvement in his other ocular symptoms.

**Conclusion:**

Our case highlights a rare constellation of a pituitary adenoma with CS invasion complicated by PA and CSS during HD. The pathophysiology of PA is not well understood, and there are very limited data regarding PA in patients with end-stage renal disease (ESRD) on HD. Prompt recognition of PA in a patient presenting with CSS, particularly in the HD setting, is essential to ensure appropriate care is provided for this medical emergency.

## 1. Introduction

Pituitary apoplexy (PA) refers to the instantaneous destruction of the pituitary gland secondary to ischemia and/or bleeding into the normal pituitary, or within a pituitary adenoma. It is a medical emergency that can have catastrophic outcomes, including acute pituitary hormonal insufficiency with hypotension and shock, acute encephalopathy and obtundation, vision loss, and cavernous sinus syndrome (CSS) [1]. CSS describes a constellation of findings due to compression of structures in the fixed space of the cavernous sinus (CS), causing multiple cranial neuropathies. Etiologies include inflammation, trauma, and neoplasms (including pituitary adenomas). CSS manifests as ophthalmoplegia due to oculomotor (CN III), trochlear (CN IV) and/or abducens (CN VI) nerve compression, facial sensory loss due to involvement of CN V1 and V2, and/or Horner's syndrome due to oculosympathetic fiber involvement. Furthermore, chemosis and proptosis can also occur due to vascular engorgement in the CS [2, 3]. We describe a unique case of an undiagnosed pituitary tumor manifesting as abrupt PA and CSS during hemodialysis (HD). The literature on PA, CSS, and the occurrence of these phenomena in patients with end-stage renal disease (ESRD) on HD is explored, including the predisposing factors, outcomes, and prognosis in such cases.

## 2. Case Presentation

**Figure 1:**
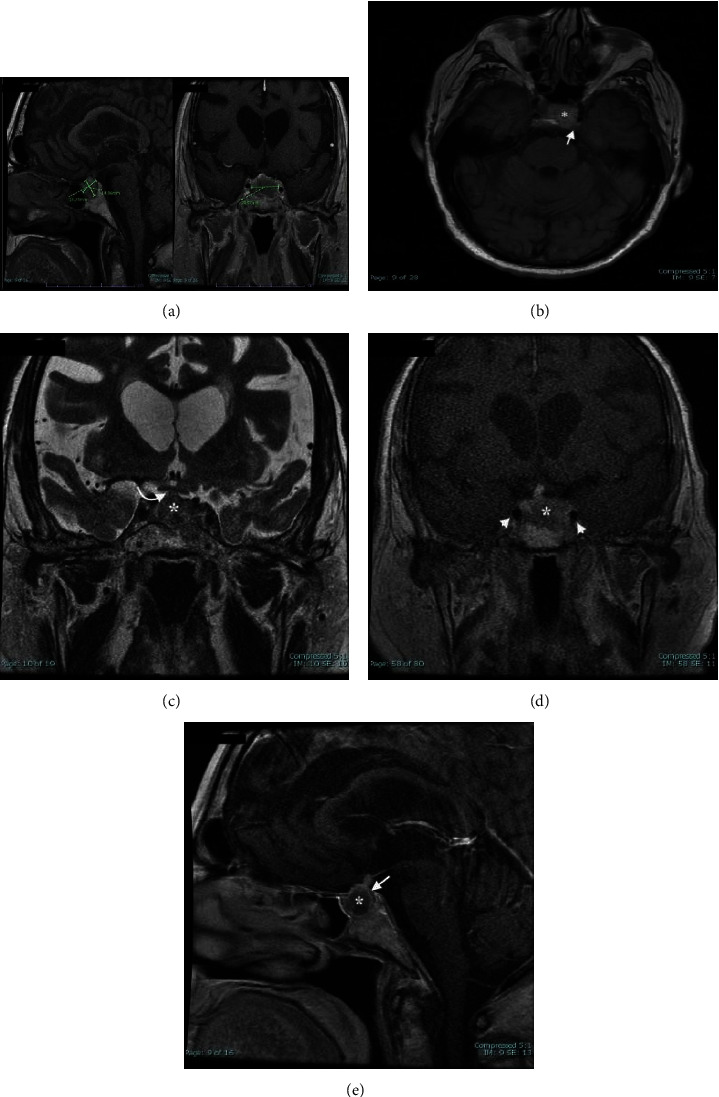
MRI brain images. (a) Sagittal and coronal T1 weighted sequence of the sellar region demonstrates a 2.5 cm × 1.5 cm × 1.5 cm heterogeneously T1 hyperintense mass expanding the sella turcica. Note the patchy T1 bright signal throughout the mass, consistent with subacute hemorrhage. (b) Axial T1 sequence of the brain again demonstrates the heterogeneously T1 hyperintense mass (asterisk) extending in to the left greater than right cavernous sinuses (arrow). (c) Coronal T2 sequence shows the T2 hypointense mass (asterisk) with minimal mass effect upon the optic chiasm (curved arrow). (d) Dynamic coronal contrast sequences show the hypoenhancing left sellar mass (asterisk). Note the preserved carotid flow voids within the cavernous sinus (arrowheads). (e) Delayed sagittal contrast sequence shows the hypoenhancing mass (asterisk) with claw-like hyperenhancing pituitary parenchyma along the periphery (arrow).

A 77-year-old male with coronary artery disease, ESRD, type 2 diabetes, and hypertension experienced sudden onset of severe headache, double vision, and left ptosis during HD. His systolic blood pressure was 90 mm Hg. HD was stopped immediately, and he was transported to a local emergency department. Upon arrival, his blood pressure (BP) was 90/55 mm Hg. MRI of the brain (Figures 1(a)–1(e)) revealed a 2.5 × 1.5 × 1.8 cm mass in the sella turcica consistent with a pituitary macroadenoma with apoplexy. There was significant invasion into the left CS, and the tumor was compressing the left prechiasmatic optic nerve.

A multiplanar, multiecho magnetic resonance image (MRI) of the brain and pituitary gland was obtained with and without gadolinium contrast utilizing a 3-Tesla MR imaging system. The examination demonstrated a 2.5 cm × 1.5 cm × 1.5 cm (TV × AP × CC) heterogeneously T1 hyperintense, T2 hypointense mass arising from the sella with expansion of the sella turcica. There is mild suprasellar extension of the mass with thickening of the pituitary infundibulum and with compression of the left prechiasmatic optic nerve. The mass invades the left greater than right cavernous sinus with preservation of the cavernous internal carotid artery flow voids. On precontrast imaging, scattered internal T1 hyperintense signal is noted throughout the mass, compatible with early subacute hemorrhage. On dynamic contrast imaging, the mass demonstrates homogeneous hypoenhancement. The constellation of these findings and enhancement patterns is compatible with a pituitary macroadenoma with superimposed pituitary apoplexy.

The patient was treated with 100 mg of intravenous hydrocortisone and intravenous fluids, and then transferred to our hospital for further management.

The patient's home medications included aspirin, atorvastatin, calcium acetate, metoprolol, insulin glargine, amlodipine, hydralazine, and famotidine. On physical examination, he was well-virilized and did not appear Cushingoid or acromegalic. His BP was 137/62 mm·Hg, heart rate was 84 beats per minute, oral temperature was 36.5°C, respiratory rate was 15 breaths/minute, and oxygen saturation was 97% on room air. Neurologic exam revealed complete ptosis of the left eye, minimal abduction and absence of other left extraocular movements, a fixed and dilated left pupil with abnormal afferent pupillary response, reduced left visual acuity of 20/100, and reduced sensation over the CN V1 and V2 distribution of the left side of the face. These findings were consistent with complete palsies of the left CNs III, IV, V1, V2, and partial palsy of left CN VI. Visual fields were grossly full to confrontation. Muscle tone, strength, and coordination were intact. The funduscopic examination was unremarkable.

The results of the admission laboratory evaluation are shown in Table 1. This revealed central adrenal insufficiency, central hypogonadism, growth hormone deficiency, and hypoprolactinemia.

**Figure 2:**
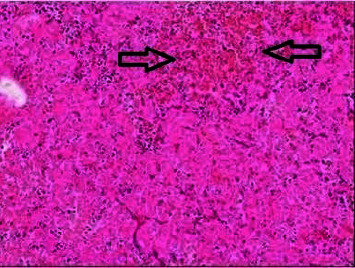
Histopathology. Extensive infarction of a pituitary adenoma with extravasation of erythrocytes consistent with hemorrhagic infarct (arrows) (H and E original magnifications ×100).

Due to the acute reduction in left visual acuity and ophthalmoplegia, the patient was advised to undergo endoscopic endonasal transsphenoidal resection of the pituitary tumor. The patient was given 100 mg of intravenous hydrocortisone before the surgery. During the procedure, there was an immediate release of the tumor contents upon incision of the dura mater, which was under significant pressure. The tumor had extended widely into the left CS and required extensive decompression. Near-total resection of the tumor was achieved. Histopathology confirmed a pituitary adenoma with hemorrhage (Figure 2). Immunohistochemistry could not be performed due to the extensive hemorrhagic necrosis.

The patient had an uneventful postoperative course, but his ocular symptoms did not improve. His free thyroxine level was low at 0.6 ng/dl (normal 0.7–1.9) on postop day 2 (Table 1), and levothyroxine 25 mcg daily was initiated. Hydrocortisone (HC) was changed to the oral route and tapered to 10 mg in the morning and 5 mg in the evening. The patient was discharged on hospital day 6 with instructions to continue these medications and follow up in the outpatient clinic in 2 weeks.

The patient failed to present for his outpatient follow-up appointments. Two months later, he was readmitted to our hospital with weakness, anorexia and vomiting in the setting of missing HD sessions for one week, and not taking the evening doses of hydrocortisone. His BP was 130/80 mm Hg. The ophthalmological examination revealed left eye ptosis with reduced extraocular movement and reduced left visual acuity. The serum cortisol was 7.6 mg/dl at 6:00 am, and the free thyroxine was normal (Table 1). HD was resumed, and he was given intravenous hydrocortisone 25 mg every 12 hours for one day. He was discharged on prednisone 5 mg daily and levothyroxine 25 mcg daily, and he was counseled regarding the importance of adherence with his prescribed medications. The patient continued to be nonadherent with outpatient follow-up. Two months later, he was admitted to an outside hospital for septic shock due to pneumonia. Stable left ocular paresis and decreased left visual acuity were again noted. The patient passed away after a prolonged hospital course.

## 3. Discussion

PA can be the first manifestation of an undiagnosed pituitary tumor. The prevalence of PA in a recent review was 6.2 cases per 100,000 people [1]. It has been hypothesized that pituitary tumors are vulnerable to hemorrhage and/or infarction leading to PA due to their extensive vascularity, increased metabolic demand, and/or compromise of the vascular supply to large tumors [4]. In a retrospective cohort analysis of patients with pituitary adenomas, two hundred eighty-eight patients were divided into pituitary hemorrhagic and nonhemorrhagic groups. In the hemorrhagic group (*n* = 81), four patients had ESRD and were receiving HD. The analysis implicated HD, and anticoagulation during HD, as potential risk factors for pituitary hemorrhage [5]. Other risk factors described in the literature include major surgery, larger macroadenoma size, gonadotropin-releasing agonist or dopamine agonist treatment, dynamic stimulation testing such as corticotropin releasing hormone or thyrotropin releasing hormone testing, and malignant disease [5]. Additionally, CS invasion is a risk factor for recurrent apoplectic events, especially in patients with residual pituitary tumor after surgery [6].

The occurrence of cerebral hemorrhage in patients on chronic dialysis has been explicated in the literature [7]. Shimokawa et al. described a case of spontaneous epidural hematoma in a patient undergoing HD. The patient developed spinal cord compression and required emergent surgery [8]. The proposed etiologic factors for intracranial hemorrhage in ESRD patients receiving HD include the uremic state itself, the use of systemic or intradialytic heparin, the use of aspirin, and sudden hemodynamic fluctuations during HD [9]. The pathophysiology of uremic bleeding is multifactorial and involves platelet dysfunction due to uremic toxins. HD reduces the platelet abnormalities due to uremia and reduces but does not eliminate the risk of hemorrhage [10].

Aspirin or heparin are used during HD to inhibit thrombosis of HD vascular access but may also increase the risk of bleeding in HD patients [9, 11]. Use of these medications has been explored as a risk factor for pituitary hemorrhage and PA in case reports. Swaid et al. reported a case of a 65-year-old female with normal renal function and no known history of pituitary disease, who developed PA with left CN III palsy shortly after aspirin and intravenous heparin in the setting of coronary catheterization. MRI brain revealed a large pituitary tumor. The patient underwent uneventful transsphenoidal pituitary surgery, although there was no recovery of CN III palsy [12]. Nagarajan et al. also described a case of PA with ophthalmoplegia after the simultaneous use of aspirin, heparin, and clopidogrel. The patient had partial ophthalmologic recovery four weeks after surgical treatment [13]. Hemodynamic and intracranial pressure fluctuations during HD are potential additional contributors to an increased risk of PA in patients with ESRD [14].

Acute hypopituitarism is commonly seen in patients with PA, with one or more anterior pituitary deficiencies often present at onset. Corticotropin deficiency is the most common, occurring in 50–80% of patients [1]. If not recognized and treated promptly, corticotropin deficiency can lead to hemodynamic instability and death. Because of this, empiric high dose glucocorticoids are recommended when PA is suspected. Other hormone deficiencies seen in PA include growth hormone deficiency (in almost all patients), thyrotropin deficiency (in 30–70% of patients), and gonadotropin deficiency (in 40–75% of patients) [1]. PA can lead to hypoprolactinemia, which is seen in 10–40% of cases [1]. Our patient had notably low prolactin levels despite having a history of ESRD, which can result in elevated prolactin levels due to both decreased renal clearance and increased production [15]. Diabetes insipidus is rare in PA, seen in less than 5% of patients [1].

The CS is a dural venous sinus and fixed space containing the internal carotid artery, the carotid sympathetic plexus, CN III, IV, V1, V2, and VI. CSS is a constellation of signs and symptoms that occur due to impaired function of two or more CNs with or without involvement of the oculosympathetic fibers running through the CS. It can be caused by vascular, inflammatory, or neoplastic (including pituitary adenomas) pathologies [3, 16].

PA can be managed surgically or conservatively. According to available guidelines, CN palsy is not an indication for surgical decompression of tumor, unless associated with reduced visual acuity, as was seen with our patient [17, 18]. Almeida et al. retrospectively compared the outcomes including CN palsy in patients with PA managed surgically versus conservatively. The recovery of CN function was not statistically different between groups. The CN improvement rate was 75% in the surgery group and 58.3% in the conservative group; however, in the conservative management group, only five out of eighteen patients had ophthalmoplegia, whereas in the surgical group, twenty-seven out of forty-nine patients had CN involvement [19].

With some similarity to our case, De La Torre et al. reported a case of PA with an isolated incomplete CN III palsy during the second hour of HD. The patient's symptoms improved temporarily with analgesic and intravenous hydration. However, the symptoms recurred and MRI brain demonstrated PA. The patient declined surgery and was treated with steroids. Ten months later, his ocular symptoms resolved with conservative management [20]. Lee et al. described a patient with a pituitary macroadenoma and ESRD on HD who developed PA and complete right eye ophthalmoplegia one day after a session of HD. Unlike our case, though, this patient also had posterior reversible encephalopathy syndrome simultaneously with PA. The MRI brain showed pituitary tumor with hemorrhage and vasogenic edema. The patient refused surgery and his ocular symptoms gradually improved with conservative treatment [21].

Patients with OP that involves a single CN have a better likelihood of recovery than those with involvement of multiple CNs, regardless of management (conservative or surgical). Moreover, age, comorbidities, and severity of the initial ocular insult play a role in successfully achieving neurological recovery [19, 22]. Our patient was an elderly man with significant comorbidities. His pituitary tumor had invaded the CS extensively, resulting in the compression of multiple CNs during the apoplectic event. Our patient underwent urgent surgical intervention due to the acute reduction in left sided visual acuity, and his vision was salvaged by preserving the optic nerve. However, despite timely surgical intervention and CS decompression, the ophthalmoplegia did not improve six months after surgery. Unfortunately, our patient did not survive beyond six months to see if his ocular symptoms might show improvement after this time.

## 4. Conclusion

We describe the case of an elderly man without a known diagnosis of a pituitary tumor who developed PA with CSS during a session of HD. Several factors likely contributed to the acute apoplectic event including uremia, hemodynamic fluctuations, and the use of anticoagulant medications (aspirin and heparin). While cases of CSS secondary to PA have been described [3], to the best of our knowledge, we report the first case of CSS due to PA in a patient with ESRD during an HD treatment session.

The risk of PA and CSS appears to be increased in patients with pituitary tumors with ESRD undergoing HD. Clinicians must be aware of this rare complication to promptly recognize symptoms suggestive of PA and/or CSS, obtain an appropriate workup with hormonal labs and pituitary MRI, and initiate treatment promptly with hormone replacement and neurosurgical consultation as indicated.

Please note that this case report has been published as a conference “abstract” only in AACE Endocrine Practice.

## Figures and Tables

**Table 1:** Laboratory results.

Laboratory test	Reference range	On admission	Postoperative day two	Postoperative day sixty
Sodium (mmol/l)	135–145	135	**132**	136
Potassium (mmol/l)	3.2–5.1	4.1	4.2	3.8
Creatinine (mg/dl)	0.72–1.25	**3.15**	**2.46**	**5.59**
ACTH (pg/ml)	7.2–63	**7.0**		
Cortisol (*μ*g/dl)	3.7–19.4	**2.1**		7.6
TSH (mIU/l)	0.350–4.700	**0.053**		
FT4 (ng/ml)	0.7–1.9	0.9	**0.6**	1.0
TT3 (ng/ml)	79.0–149	**37.3**		
Prolactin (ng/ml)	2.6–18.1	**1.9**		5.1
IGF-1 (ng/ml)	37–219	**16**		
FSH (mIU/l)	1.4–13.6	5.25		
LH (mIU/l)	0.6–12.1	2.4		
Total testo (ng/dl)	240–950	**<7.0**		

Abnormal values are bolded. ACTH: adrenocorticotropic hormone, TSH: thyroid-stimulating hormone, FT4: free thyroxine, TT3: total triiodothyronine, IGF-1: insulin-like growth factor-1, (age and sex matched range), FSH: follicle-stimulating hormone, LH: luteinizing hormone, and Total testo: total testosterone.

## Data Availability

The data supporting the finding of this case are available within the article.
